# Targeting c-Myc Unbalances UPR towards Cell Death and Impairs DDR in Lymphoma and Multiple Myeloma Cells

**DOI:** 10.3390/biomedicines10040731

**Published:** 2022-03-22

**Authors:** Andrea Arena, Maria Anele Romeo, Rossella Benedetti, Maria Saveria Gilardini Montani, Mara Cirone

**Affiliations:** Department of Experimental Medicine, Sapienza University of Rome, Viale Regina Elena 324, 00161 Rome, Italy; a.arena@uniroma1.it (A.A.); mariaanele.romeo@uniroma1.it (M.A.R.); rossella.benedetti@uniroma1.it (R.B.); mariasaveria.gilardinimontani@uniroma1.it (M.S.G.M.)

**Keywords:** multiple myeloma (MM), primary effusion lymphoma (PEL), c-Myc, IRE1 α/XBP1, CHOP, DDR

## Abstract

Multiple myeloma (MM) and primary effusion lymphoma (PEL) are aggressive hematological cancers, for which the search for new and more effective therapies is needed. Both cancers overexpress c-Myc and are highly dependent on this proto-oncogene for their survival. Although c-Myc inhibition has been shown to reduce PEL and MM survival, the underlying mechanisms leading to such an effect are not completely clarified. In this study, by pharmacologic inhibition and silencing, we show that c-Myc stands at the cross-road between UPR and DDR. Indeed, it plays a key role in maintaining the pro-survival function of UPR, through the IRE1α/XBP1 axis, and sustains the expression level of DDR molecules such as RAD51 and BRCA1 in MM and PEL cells. Moreover, we found that c-Myc establishes an interplay with the IRE1α/XBP1 axis whose inhibition downregulated c-Myc, skewed UPR towards cell death and enhanced DNA damage. In conclusion, this study unveils new insights into the molecular mechanisms leading to the cytotoxic effects of c-Myc inhibition and reinforces the idea that its targeting may be a promising therapeutic approach against MM and PEL that, although different cancers, share some similarities, including c-Myc overexpression, constitutive ER stress and poor response to current chemotherapies.

## 1. Introduction

c-Myc proto-oncogene is a transcription factor with a key role in the control of essential biological processes such as cell proliferation, metabolism and cell death. c-Myc overexpression is frequently observed in solid as well as hematological cancers, including primary effusion lymphoma (PEL), a B cell lymphoma linked to Kaposi’s sarcoma-associated herpesvirus (KSHV), and multiple myeloma (MM), a plasma cells malignancy, although mutations or translocations usually do not occur in these cancers [1,2]. Given that c-Myc overexpression is known to drive cancer cell proliferation, several inhibitors of this transcription factor have been developed and some of them have been also introduced in pre-clinical trials against cancers known to be c-Myc-addicted. Both PEL and MM are among cancers highly dependent on c-Myc for their survival and growth [3,4]. Interestingly, we have recently shown that the reduction of c-Myc expression and the concomitant wtp53 activation was one of the mechanisms through which DNA damage, induced by PARPs and CHK1 inhibition, promoted PEL cell death [5]. Interestingly, in PEL cells that harbor KSHV in a latent state, c-Myc overexpression has been reported to contribute to the maintenance of viral latency [6] while, on the other hand, the activation of wtp53/p21 axis has been shown by ours and other’s laboratories to trigger viral replication [7,8,9]. c-Myc inhibitors have been shown to be a promising therapy also against MM, both as single agents [10] and in combination with other drugs such as tyrosine kinase inhibitors [11].

Targeting c-Myc has been shown to interrupt its positive interplay with IRE1α/XBP1 [12], a pathway of UPR mainly involved in survival of cancer cells in the course of basal or induced ER stress [13,14]. Moreover, XBP1s has been shown to be necessary for optimal c-Myc mRNA and protein expression in prostate cancer [15]. c-Myc may also induce an optimal expression of ATF4 and cooperate with it in the regulation of a specific program of c-Myc target genes [16]. This evidence suggests that UPR sensors and c-Myc are strongly interconnected and together control cancer cell survival/proliferation [17].

Interestingly, previous studies have indicated that the IRE1α/XBP1 axis is able to sustain the expression of several DDR molecules, either involved in homologous repair (HR) and in non-homologous end joining (NHEJ) pathway [18]. Moreover, c-Myc has been reported to directly control the expression of HR molecules such as BRCA1 [19,20], suggesting that this molecule can control DNA damage repair both directly and indirectly. Based on this knowledge, in this study we investigated the impact of pharmacological inhibition or silencing of c-Myc on the dysregulation of UPR as well as DDR, adaptive responses essential for cell survival particularly of cancers such as PEL and MM characterized by c-Myc overexpression and a constitutive high level of ER stress [21,22].

## 2. Materials and Methods

### 2.1. Cell Cultures and Treatments

MM cell lines SKO-007 (J3) (SKO) and RPMI-8226 (RPMI) and PEL cell lines BC3 and BCBL1 [23] were maintained in RPMI 1640 medium (Sigma-Aldrich, Burlington, MA, USA), supplemented with 10% fetal bovine serum (FBS) (Sigma-Aldrich, Burlington, MA, USA), L-glutamine (2 mM) (Aurogene, Rome, Italy), streptomycin/penicillin (100 μg/mL) (Aurogene, Rome, Italy) at 37 °C and 5% CO_2_ humidified atmosphere. The cells were seeded into 6-well plates at a density of 6 × 10^5^ cells per well in a final volume of 2 mL. Subsequently, the cells were treated for 24 h (h) with c-Myc Inhibitor (I c-Myc) (50 μM) (Sigma-Aldrich, Burlington, MA, USA, 475956) or IRE1 RNAse inhibitor (4µ8c) (20 μM) (Sigma-Aldrich, Burlington, MA, USA, SML0949). Untreated cells were used as a control group (CTRL).

### 2.2. c-Myc Silencing

RPMI and BC3 cells were seeded into 6-well plates at a density of 6 × 10^5^ cells per well and transfected with c-Myc siRNA (si c-Myc, Santa Cruz Biotechnology Inc., Dallas, TX, USA, sc-29226), for knockdown, or Control siRNA-A (scramble, SCR, Santa Cruz Biotechnology Inc., Dallas, TX, USA, sc-37007) by using INTERFERin^®^ reagent (Polyplus-transfection, Illkirch-Graffenstaden, France) in accordance with the manufacturer′s protocol. The cells were collected after 48 h of transfection for subsequent analysis.

### 2.3. Trypan Blue Exclusion Assay

Following treatments, as above reported, the Trypan Blue (Sigma-Aldrich, Burlington, MA, USA) dye exclusion assay was used for viable cell counting. Live cells were counted by light microscopy using a Neubauer hemocytometer. The experiments were performed in triplicate and repeated at least three times. 

### 2.4. Western Blot Analysis

To evaluate protein expression, we performed Western blot analysis of the cells harvested after treatment, centrifuged at 1200 rpm for 5 min (min) at room temperature (RT) in phosphate-buffered saline (PBS) and subsequently lysed in RIPA buffer (150 mM NaCl, 1% NP-40, 50 mM Tris-HCl (pH 8), 0.5% deoxycholic acid, 0.1% SDS, protease and phosphatase inhibitors). Cell lysates were centrifuged for 45 min at 14,000 rpm, 4 °C to remove cellular debris. Total protein concentration was determined in supernatant by Quick Start Bovine Serum Albumin (BSA) assay (BIO-RAD laboratories, Hercules, CA, USA). Then, 15 µg of protein was denatured in loading buffer by heating for 10 min at 70 °C, loaded and separated by electrophoresis on 4–12% NuPage Bis-Tris gels (Life Technologies, Carlsbad, CA, USA), as reported before [24]. Briefly, the proteins in gel were transferred to nitrocellulose (NT) membranes (BIO-RAD laboratories, Hercules, CA, USA) for 1 h in Tris-Glycine buffer. NT membranes were first blocked with 3% BSA (SERVA, Reno, NV; 11,943.03) in 1 × PBS-0.1% Tween20 for 1 h at RT and then probed with specific antibodies. After 3 washes with 1 × PBS-0.1% Tween 20, the membranes were incubated with secondary antibody for 1 h at RT. NT membranes were further washed in 1 X PBS-0.1% Tween20 and then developed using ECL Blotting Substrate (Advansta, San Jose, CA, USA).

### 2.5. Antibodies

The antibodies used to identify specific proteins in Western blot are listed as follows: rabbit polyclonal anti-PARP (1:500) (Cell Signaling, Danvers, MA, USA, 9542), mouse monoclonal anti-caspase-3 (1:100) (clone E-8) (Santa Cruz Biotechnology Inc., Dallas, TX, USA, sc-7272), rabbit polyclonal anti-XBP1 (1:1000) (Novus Biologicals, Littleton, CO, NB100-80861), rabbit polyclonal anti-CHOP (GADD153) (1:1000) (Proteintech, Rosemont, IL, USA, 15204-1-AP), rabbit polyclonal anti-phospho-EIF2α (Ser51) (1:1000) (Cell Signaling, Danvers, MA, USA, 9721), rabbit polyclonal anti-EIF2α (1:4000) (Cell Signaling, Danvers, MA, USA, 9722), mouse monoclonal anti-γH2AX (phospho-Ser 139) (1:100) (Santa Cruz Biotechnology Inc., Dallas, TX, USA, sc-517348), mouse monoclonal anti-BRCA1 (1:1000) (EMD Millipore, Burlington, MA, OP92), mouse monoclonal anti-RAD51 (1:200) (Santa Cruz Biotechnology Inc., Dallas, TX, USA, sc-377467), rabbit polyclonal anti-c-MYC (1:500) (Proteintech, Rosemont, IL, USA, 10828-1-AP). Mouse monoclonal anti-β Actin (1:10,000) (Sigma-Aldrich, Burlington, MA, USA) was used as loading control. The goat anti-mouse IgG-HRP (1:30,000) (Bethyl Laboratories, Montgomery, TX, USA, A90-116P) and goat anti-rabbit IgG-HRP (1:30,000) (Bethyl Laboratories, Montgomery, TX, USA, A120-101P) were used as secondary antibodies. All the primary and secondary antibodies were diluted in 1 X PBS-0.1% Tween20 solution containing 3% of BSA (SERVA, Reno, NV; 11,943.03).

### 2.6. Indirect Immunofluorescence Assay (IFA)

Indirect immunofluorescence assay for γH2AX was performed on SKO and BC3 cells, after treatment with I c-Myc (50 µM), to evaluate foci formation. Briefly, after 24 h of treatment, the cells were washed with PBS, applied onto multispot microscope slides and air-dried. The samples were then incubated with 2% paraformaldehyde (Electron Microscopy Science) for 30 min and then permeabilized with 0.1% Triton X-100 (Sigma-Aldrich, Burlington, MA, USA) for 5 min. After 3 washes, cells were incubated with 1% glycine, 3% BSA (SERVA) for a further 30 min. Then cells were incubated with the primary monoclonal antibody against γH2AX (phosphor-Ser 139) (1:100 in PBS) (Santa Cruz Biotechnology Inc., Dallas, TX, USA, sc-517348) for 1 h at RT. Slides were then washed 3 times with PBS and cells were further incubated with a polyclonal conjugated-Cy3 sheep anti-mouse antibody (1:2000 in PBS) (Jackson ImmunoResearch, UK) for 30 min at RT. After 3 washes in PBS, cells were stained with DAPI (1:5000 in PBS) (Sigma-Aldrich, Burlington, MA, USA) for 1 min at RT. Slides were further washed in PBS, mounted with glycerol:PBS (1:1) and analyzed with an Apotome Axio Observer Z1 inverted microscope (Zeiss, Germany) equipped with an AxioCam MRM Rev.3 (Germany) at 40 magnification. Foci amount per cell was counted by Image J software (USA).

### 2.7. RNA Isolation and Quantitative Real Time Polymerase Chain Reaction (qRT-PCR)

Total RNA from RPMI and BC3 treated with I c-Myc (50 μM) was isolated with TRIzol™ Reagent (Invitrogen, Life Technologies Corporation, Carlsbad, CA, USA) according to the manufacturer’s instructions [25]. The concentration and purity of RNA were determined at 260/280 nm using a Nanodrop (MaestroNano Micro-Volume Spectrophotometer, MaestroGen). *BRCA1* and *RAD51* mRNA expression levels were analyzed using TaqMan gene expression assays (Applied Biosystems, Vilniaus, Lithuania). Briefly, 2 µg of total RNA was reverse-transcribed into cDNA using High-capacity cDNA Reverse Transcription Kit (Thermo Fisher Scientific, Waltham, MA, USA) according to the manufacturer’s instructions. For each PCR, a mastermix was prepared on ice, containing per sample: 2 µL cDNA (20 ng), 1 µL of TaqMan gene expression assays specific for each mRNA analyzed (*BRCA1* and *RAD51*) (Applied Biosystem, Vilniaus, Lithuania, HS01556193-m1 and HS00153418) and 10 µL of 2× TaqMan Fast Advance Master Mix. The PCRs were run on an Applied Biosystem Real-Time thermocycler. Each amplification was performed in triplicate, and the average of three threshold cycles was used to calculate transcript abundance. The starting concentration of each specific product was divided by the geometric mean of the starting concentration of reference genes (*GAPDH* and *B2M*) (Applied Biosystem, Vilniaus, Lithuania, HS99999905-m1 and HS99999907-m1) and this ratio was compared between treated/control groups.

### 2.8. Densitometric Analysis

The quantification of protein bands was performed by densitometric analysis using the Image J software (1.47 version, NIH, Bethesda, MD, USA), which was downloaded from the NIH website (http://imagej.nih.gov (accessed on 10 February 2022)).

### 2.9. Statistical Analysis

Results are represented by the mean ± standard deviation (S.D.) of at least three independent experiments and statistical analyses were performed with Graphpad Prism^®^ software (Graphpad software Inc., La Jolla, CA, USA). Two-tailed Student’s *t*-test were used to demonstrate statistical significance. Difference was considered as statistically significant when *p*-value was: * < 0.05; ** < 0.01; *** < 0.001 and **** < 0.0001

## 3. Results

### 3.1. c-Myc Inhibition Triggers an Apoptotic Cell Death in MM and PEL Cell Lines

**Figure 1:**
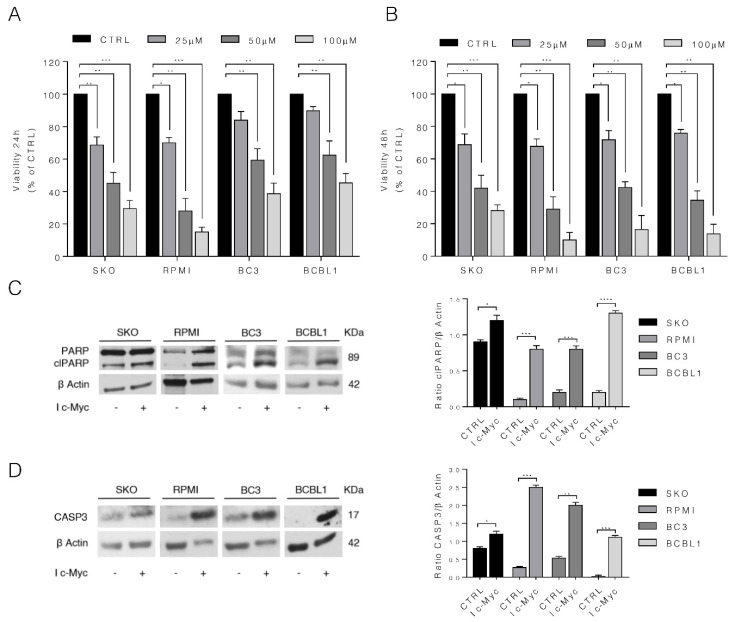
c-Myc inhibition induces apoptosis in MM and PEL cell lines. SKO and RPMI (MM) cells and BC3 and BCBL1 (PEL) cells were cultured with different doses (25, 50 and 100 µM) of c-Myc inhibitor (I c-Myc); (**A**,**B**) cell viability was evaluated by Trypan Blue exclusion assay after 24 h and 48 h of treatments. The histograms represent the mean plus S.D. of live cells as percent of untreated control cells, * *p* value: * < 0.05; ** < 0.01; *** < 0.001; (**C**,**D**) Protein expression level of cleaved PARP (clPARP) and caspase3 (CASP3) was evaluated by Western blot analysis in all four lines treated with 50 µM I c-Myc for 24 h. β Actin was used as loading control and one representative experiment is shown. The histograms represent the densitometric analysis of the ratio of clPARP/β Actin and CASP3/β Actin of three different experiments. Data are represented as the mean plus S.D. *p* value: * < 0.05; ** < 0.01; *** < 0.001; **** < 0.0001.

We exposed SKO and RPMI multiple myeloma cells and BC3 and BCBL1 PEL cells to c-Myc inhibitor (I c-Myc) as their cell growth is known to be dependent on c-Myc overexpression [3,4]. As shown in Figure 1A,B, c-Myc inhibitor, which acts by interfering with c-Myc/Max interaction and thus preventing c-Myc target gene expression, reduced cell survival in a dose- and time-dependent fashion in all MM and PEL cell lines studied. To assess whether the reduction of cell survival was due to the induction of apoptosis, we evaluated PARP cleavage and caspase 3 activation. We found that PARP cleavage (Figure 1C) and caspase 3 activation (Figure 1D) increased in these cell lines, suggesting the occurrence of an apoptotic cell death in both MM and PEL cells.

### 3.2. c-Myc Inhibition, by Reducing IRE1α/XBP1s and Enhancing p-EIF2α/CHOP Axis Activation, Unbalances UPR towards Cell Death in MM and PEL Cells

**Figure 2:**
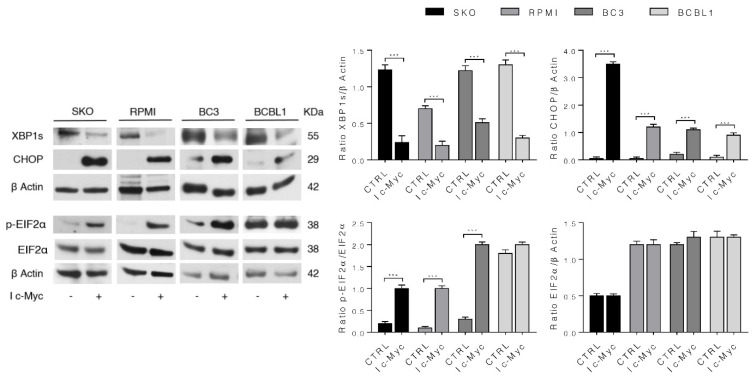
c-Myc inhibition decreases IRE1 α/XBP1s and increases p-EIF2α/CHOP axis to induce cell death in MM and PEL cells. SKO and RPMI (MM) cells and BC3 and BCBL1 (PEL) cells were cultured with 50 µM of c-Myc inhibitor (I c-Myc) for 24 h and protein expression level of XBP1s, CHOP and p-EIF2α was evaluated by Western blot analysis. β Actin was used as loading control and one representative experiment is shown. The histograms represent the densitometric analysis of the ratio of specific protein/β Actin of three different experiments. Data are represented as the mean plus S.D. *p* value: *** < 0.001.

c-Myc has been shown to activate the IRE1α/XBP1 pathway in breast cancer cells [12]. Therefore, here we investigated the expression level of spliced XBP1 (XBP1s), mediated by the activation of the endoribonuclease activity of IRE1α, in MM and PEL cells in which c-Myc was inhibited. We found that MM and PEL cells displayed a reduced XBP1s expression with a concomitant upregulation of CHOP (Figure 2). Accordingly, the PERK/EIF2α branch of UPR, the main pathway involved in CHOP upregulation, was hyper-activated following treatment by c-Myc inhibitor compared to the control cells (Figure 2). These findings suggest that c-Myc inhibition unbalances UPR towards cell death, by reducing the activation of pro-survival IRE1α/XBP1s axis and increasing the pro-apoptotic PERK/EIF2α/CHOP axis.

### 3.3. c-Myc Inhibition Increases DNA Damage by Downregulating BRCA1 and RAD51 in MM and PEL Cell Lines

**Figure 3:**
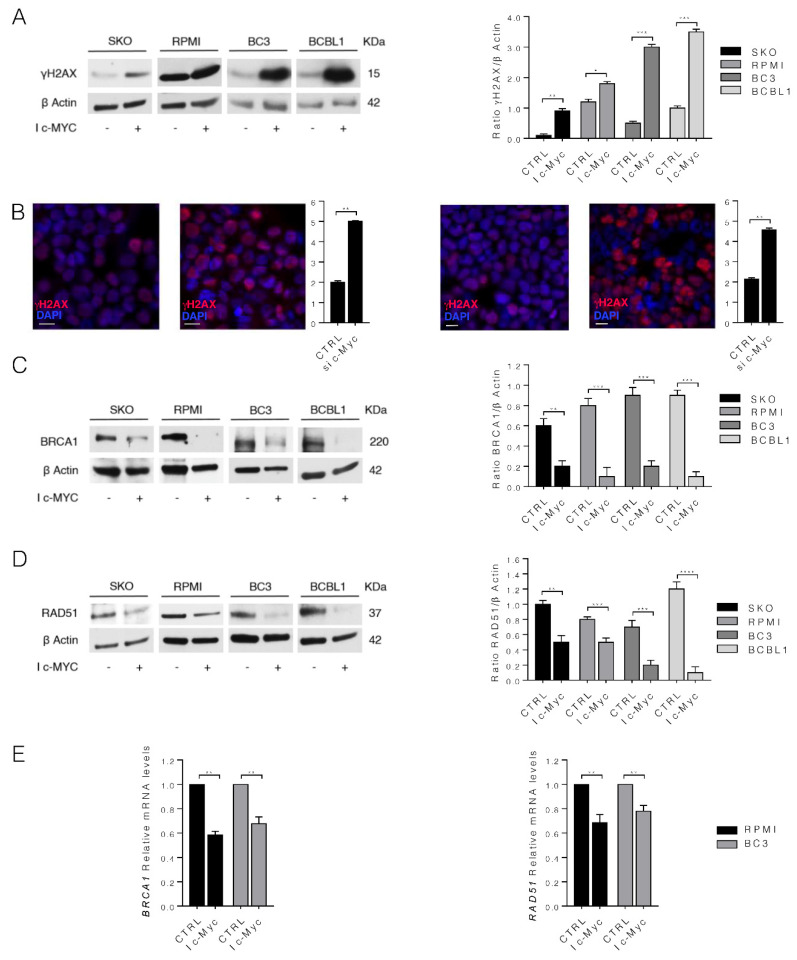
c-Myc inhibition increases DNA damage and downregulates BRCA1 and RAD51 in both MM and PEL cell lines. SKO and RPMI (MM) cells and BC3 and BCBL1 (PEL) cells were cultured with 50 µM of c-Myc inhibitor (I c-Myc) for 24 h. (**A**,**C**,**D**) Protein expression level of γH2AX, BRCA1 and RAD51 was evaluated by Western blot in all the cell lines. Untreated cells were used as control (CTRL). β Actin was used as loading control and one representative experiment is shown. The histograms represent the mean plus S.D. of the densitometric analysis of the ratio of specific protein/β Actin of three different experiments. *p* value: * < 0.05; ** < 0.01; *** < 0.001; **** < 0.0001; (**B**) γH2AX foci (red) were assessed by IFA in SKO (**left**) and BC3 (**right**) cell lines. DAPI (blue) was used for nuclear staining. The histograms represent the mean plus S.D. of the number of foci/cell from three different experiments. Bars = 10 μm. One representative experiment out of three is reported. *p* value: ** < 0.01. (**E**) qRT-PCR of BRCA1 and RAD51 in RPMI and BC3 cell lines treated with 50 µM of c-Myc inhibitor (I c-Myc) for 24 h. Data are expressed relative to the geometric mean of the starting concentration of reference genes (GAPDH and B2M). The histograms represent the mRNA expression levels of indicated genes of three different experiments. Data are represented as the mean relative to the control plus S.D. ** *p* value < 0.01.

We then evaluated whether c-Myc inhibition could have an impact on DNA damage, as XBP1s, shown to be sustained by c-Myc, has been reported to protect cells from DNA damage [26]. As shown in Figure 3A,B, H2AX phosphorylation (γH2AX) increased and a higher number of γH2AX-positive foci was also observed in both MM and PEL cell lines treated by c-Myc inhibitor, indicating increased DNA damage. We then evaluated whether the enhanced DNA damage could correlate with a reduced expression level of molecules playing a key role in HR of DNA damage. We found that BRCA1 and RAD51 expression level was reduced by c-Myc inhibition in all four cell lines studied (Figure 3C,D), suggesting that increased DNA damage induced by c-Myc inhibitor could occur in correlation with BRCA1 and RAD51 downregulation.

We then evaluated whether BRCA1 and RAD51 reduction could occur at transcriptional level. For this aim, we performed a qRT-PCR and, as shown in Figure 3E, both RAD51 and BRCA1 mRNA were reduced, suggesting that protein downregulation was due to a reduction of mRNA expression.

### 3.4. c-Myc Silencing Confirms the Role of c-Myc on MM and PEL Cell Survival and UPR and DDR Regulation

**Figure 4:**
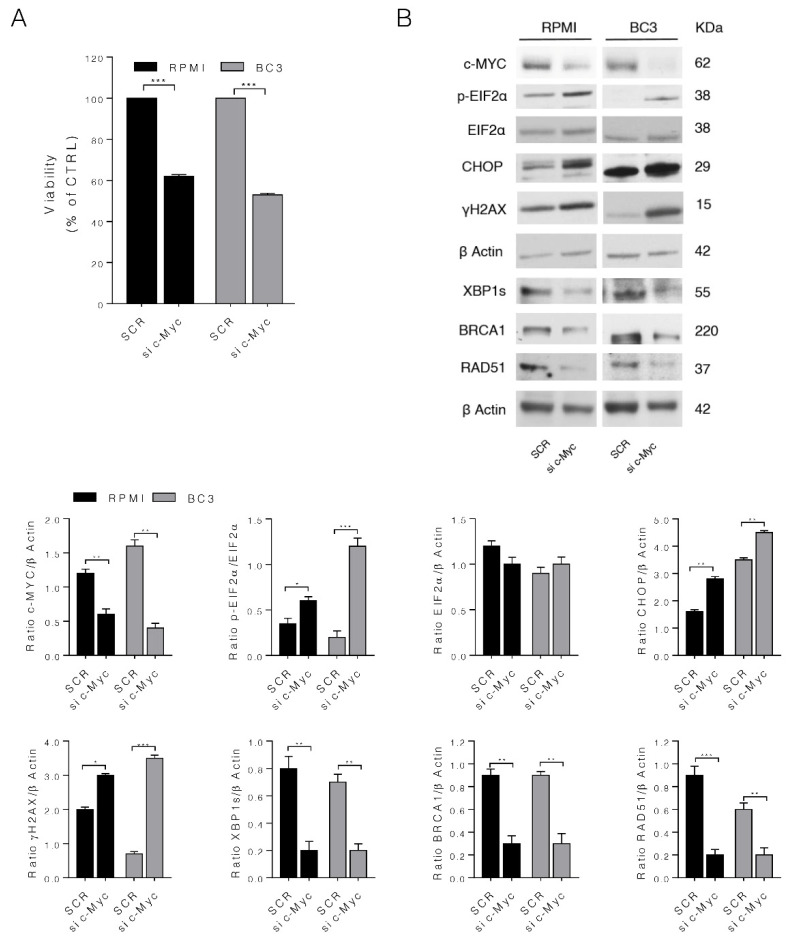
c-Myc silencing confirms the role of c-Myc on cell survival, UPR and DDR. c-Myc were silenced in RPMI (MM) and BC3 (PEL) cells for 48 h and then (**A**) cell viability was evaluated by Trypan Blue exclusion assay. The histograms represent the mean plus S.D. of live cells as percent of scramble control (SCR) cells, *** *p*-value < 0.001; and (**B**) protein expression level of specific proteins was evaluated by Western blot analysis. β Actin was used as loading control and one representative experiment is shown. The histograms represent the densitometric analysis of the ratio of specific protein/β Actin of three different experiments. Data are represented as the mean plus S.D. *p* value: * <0.05; ** < 0.01; *** < 0.001.

We then silenced c-Myc to confirm the effects observed by c-Myc pharmacological inhibition in both MM and PEL cells. First, we assessed the role of c-Myc knock-down on cell survival and then evaluated the impact of c-Myc silencing on UPR and DDR. As shown in Figure 4A, the silencing of c-Myc reduced MM and PEL cell survival and also upregulated p-EIF2α, CHOP and γH2AX while downregulating XBP1s, BRCA1 and RAD51 (Figure 4B), thus reproducing the effects induced by c-Myc pharmacological inhibition.

### 3.5. IRE1α Endoribonuclease Inhibition by 4μ8c Reduces MM and PEL Cell Survival, Downregulates c-Myc and Mimics the Effects of c-Myc Inhibition on UPR and DDR

**Figure 5:**
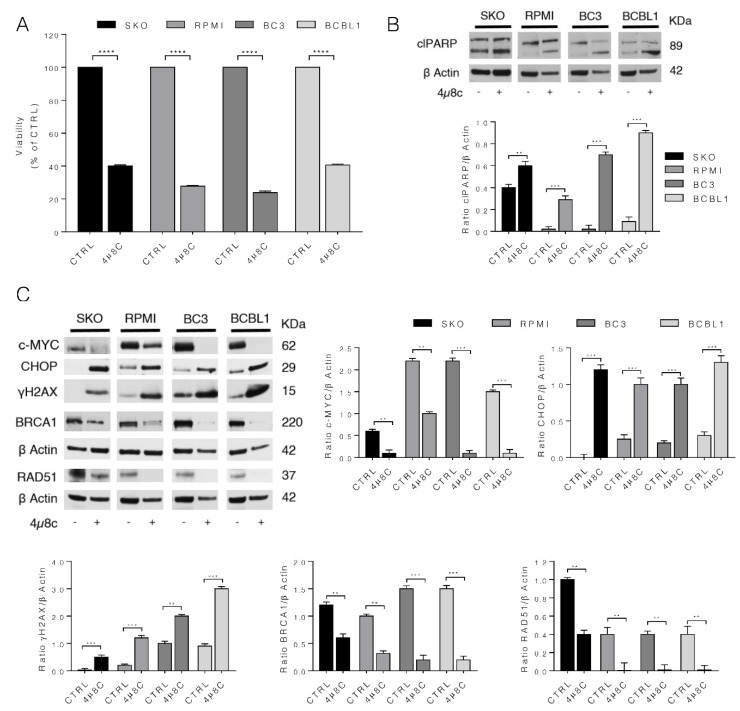
4μ8c, inhibitor of IRE1α, interferes with MM and PEL cell survival, cell death and with IRE1 α/XBP1 pathway and DDR, as observed with c-Myc inhibition. SKO and RPMI (MM) cells and BC3 and BCBL1 (PEL) cells were cultured with 20 µM of 4µ8c for 24 h and then (**A**) cell viability was evaluated by Trypan Blue exclusion assay. The histograms represent the mean plus S.D. of live cells as percent of control (CTRL) cells, **** *p*-value < 0.0001; (**B**,**C**) protein expression level of specific proteins was evaluated by Western blot analysis. β Actin was used as loading control and one representative experiment is shown. The histograms represent the densitometric analysis of the ratio of specific protein/ β Actin of three different experiments. Data are represented as the mean plus S.D. *p* value: ** < 0.01; *** < 0.001.

To explore whether the other way around could also occur and thus if IRE1α endoribonuclease inhibition could downregulate c-Myc, unbalance UPR and increase DNA damage, we treated MM and PEL cells with 4μ8c [20 μM] for 24 h, based on our previous studies [14]. 4μ8c is an inhibitor of IRE1α endoribonulease activity. As shown in Figure 5A, 4μ8c reduced cell survival in both MM and PEL cells, downregulated c-Myc, increased CHOP expression, reduced BRCA1 and RAD51 and increased γH2AX (Figure 5B). These findings suggest that XBP1s and c-Myc establish an interplay in which, by sustaining each other, control UPR and DDR in both MM and PEL cells.

## 4. Discussion

MM and PEL are hematological malignancies whose prognoses remain still poor due to their aggressive course and low responsiveness to chemotherapies. Although UPR is mainly activated as an adaptive response to help cancer cells face stressful conditions [27], ER stress and UPR may be manipulated to induce cell death. This may represent a promising therapeutic option against MM and PEL, cancers characterized by a high level of basal stress that may be exacerbated to activate the pro-death functions of UPR [28]. Due to the constitutive ER stress, treatment with proteasome inhibitors such as Bortezomib, which may exacerbate it, also represent a therapeutic option against these cancers [21,29]. Interestingly, manipulation of ER stress/UPR may also be successful in reducing cell survival against cancers carrying mutant p53 [30], as may be in the case of MM, which are known to escape apoptosis and resist to the treatment with DNA damaging agents. The option to target UPR becomes even more promising when considering that a cross-talk between UPR and DDR is clearly emerging [18,31]. In particular, it has been demonstrated by ours and other’s laboratories that ER stress, and in particular the activation of PERK arm of UPR, can reduce the expression of RAD51 and consequently increase DNA damage and cytotoxicity of DNA damaging agents [32,33]. However, the major role in the control of the expression of DDR molecules by UPR sensors seems to be played by the IRE1α XBP1 axis, as it may affect the mRNA expression and degradation of several molecules involved in both HR and NHEJ DNA repair pathways [18].

Previous studies have investigated the relationship between UPR sensors and c-Myc, showing that c-Myc activity is strongly interconnected with the activation of the IRE1α/XBP1 axis [15] or with PERK-EIF2α-ATF4, whose signaling may promote the oncogenic effect of c-Myc [34].

In this study, we found that one of the mechanisms through which c-Myc inhibition reduced cell survival of both MM and PEL cell lines was the interruption of the cross-talk that c-Myc establishes with the IRE1α/XBP1 axis, which was accompanied by the upregulation of CHOP, the downregulation of RAD51 and BRCA1 and the increase of DNA damage. The inhibition of the IRE1α/XBP1 pathway may unbalance UPR, skewing it towards cell death, as reported in previous studies [35]. c-Myc is an important therapeutic target in cancer [32,36], given that the majority of cancers are dependent on c-Myc overexpression for their growth/survival. This proto-oncogene is also overexpressed when not mutated or translocated, being upregulated downstream of oncogenic pathways such as STAT3 [37] or Wnt/B-catenin [38], which are known to be activated in cancers including PEL [39] and MM [40]. In conclusion, in this study we show that c-Myc can be considered a molecule at the cross-road between UPR and DDR in correlation with its capacity to cross-talk with the IRE1α/XBP1 arm of UPR, as observed in these hematological malignancies. c-Myc pharmacological inhibition or silencing was indeed accompanied by the unbalance of UPR towards cell death and a higher DNA damage, indicated by the increase of H2AX phosphorylation (γH2AX) and the number of γH2AX-positive foci. Therefore, targeting c-Myc may represent a promising approach against aggressive cancers such PEL and MM that, although different, share some similarities such as the presence of basal ER stress, the constitutive activation of oncogenic pathways such as STAT3 and Wnt/B-catenin and the overexpression c-Myc. Interestingly, given the impairment of DRR, the inhibition of c-Myc could also offer the opportunity to sensitize these cancer cells to the cytotoxic effect of DNA damaging agents and improve the outcome of such treatments.

## Data Availability

The datasets generated and/or analyzed during the current study are available from the corresponding author upon reasonable request.
